# Effect of Nano Ceramic Coating on Color Perceptibility and Acceptability of Polymethylmethacrylate: In Vitro and Clinical Study

**DOI:** 10.3390/ma15248748

**Published:** 2022-12-08

**Authors:** Laura Koo Min Chee, Arghya Kamal Bishal, Harshdeep Singh Bhatia, Alvin G. Wee, Christos Takoudis, Cortino Sukotjo, Judy Chia-Chun Yuan

**Affiliations:** 1 Private Practice, JBLNYC, 923 5th Avenue, New York, NY 10021, USA; 2 Intel Corporation, Hillsboro, OR 97124, USA; 3 Department of Chemical Engineering, College of Engineering, University of Illinois Chicago, 929 W. Taylor St., Chicago, IL 60608, USA; 4 Department of Restorative Sciences, School of Dentistry, University of Minnesota, 9-470 Moos Tower, 515 Delaware St. SE, Minneapolis, MN 55455, USA; 5 Department of Biomedical Engineering, College of Engineering, University of Illinois Chicago, 851 S. Morgan, Chicago, IL 60607, USA; 6 Department of Restorative Dentistry, College of Dentistry, University of Illinois Chicago, 801 S. Paulina, Chicago, IL 60612, USA

**Keywords:** poly methyl methacrylate, denture base, color, titanium dioxide, maxillofacial prosthesis, prosthesis coloring

## Abstract

The effect of a novel nano-ceramic coating (TiO_2_) using an atomic layer deposition (ALD) technique on the surface of polymethyl methacrylate (PMMA) material was investigated. The patients’ and clinicians’ perception and acceptance of the PMMA color with TiO_2_ coating were also examined. In vitro color measurement was performed on thirty specimens (light, original, and dark pink) before and after TiO_2_ coating. Patients’ and clinicians’ perception and acceptance of color changes on PMMA were measured and compared. Descriptive and analytic statistics were analyzed (a = 0.05). TiO_2_ films were successfully deposited on the PMMA specimen by the ALD technique. Color changes after TiO_2_ coating were observed on all three PMMA shades, significantly higher than the established 50:50% perceptibility threshold, but below the established 50:50% acceptability threshold. The percentage of patients that perceived a color difference after TiO_2_ coating were 83.3%, 63.9%, and 77.8% for light, original, and dark pink, respectively. The percentages of clinicians that were satisfied with the color difference were 96.4%, 80%, and 69.2% for light, original, and dark pink, respectively. Color changes after TiO_2_ coating were observed, but below the acceptable threshold. The clinical survey demonstrated that a color difference was perceived but was clinically acceptable. In general, laypeople have lower perception and higher acceptance of changes in PMMA color than clinicians.

## 1. Introduction

Rehabilitation of individuals with maxillectomies due to tumor may involve surgical reconstruction and/or prosthetic rehabilitation using obturator prostheses to restore function of speaking, chewing, and swallowing [1,2]. A maxillary obturator usually consists of an obturator bulb and a denture component. The common materials to fabricate the obturator are poly methyl methacrylate (PMMA), silicone rubber, and titanium [1].

PMMA is commonly used as denture base material for removable, dento-maxillary, maxillofacial, and implant retained/supported fixed and removable prostheses, due to its adequate strength, durability, accuracy, biocompatibility, and esthetics [3,4,5,6]. However, PMMA has poor wear resistance resulting in surface degradation and increased surface roughness [7]. PMMA is also porous, and its surface promotes initial adhesion of *Candida albicans* [8,9,10,11]. It leads to microbial attachment, colonization, and the formation of bacterial denture plaque that promotes denture stomatitis, peri-implantitis, and increased risk of developing systemic diseases including pneumonitis and systemic candidiasis. Ultimately, *Candida albicans* may lead to increased prevalence of fungal infection with obturator prostheses-wearers. These inherent, less than ideal properties have led to the advancement of these denture base materials that promote less microbial adhesion, particularly the application of surface coatings by atomic layer deposition (ALD). ALD is a growth technique that deposits precise nano-thin films of metal oxides on both external and internal particle surfaces [12,13,14,15,16]. Additional advantages of ALD include independence of line of sight and facilitation of chemical bonding between the coating material and specimen [12,13,14,15,16,17].

Titanium dioxide (TiO_2_) is a non-toxic photocatalyst initially used as environmental purification material, and later used for application in pharmaceutical, cosmetic industries, and medical devices [18,19]. The development of a TiO_2_ film has shown multiple effects, benefits, and applications for PMMA [20,21,22]. TiO_2_ coating has been successfully applied to PMMA denture base surface with the ALD technique at 65 °C. A 30 nm TiO_2_ coating was shown to decrease water contact angle and reduce *Candida. albicans* attachment by 63–77%, without change in flexural strength (MPa) of PMMA material [23]. Moreover, 30 nm thickness of TiO_2_ film provided a stable adherent film that was unaffected by brushing test and denture cleanser sonication for 1 h has been reported [23]. Despite the beneficial photocatalytic properties, the coating is white (transparent-whitish) in color, which can potentially influence the color of the acrylic denture base material [24]. However, TiO_2_ coating was shown to slow down the process of color change of heat-cured acrylic resin stored in different beverages [25]. Limited evidence exists regarding color changes of PMMA with this TiO_2_ coating [24,25], particularly no report of human subjects’ perception and acceptance of the TiO_2_ coated PMMA color.

Color is a complex science, as the perception of color is a subjective experience creating challenges in color measurements. The three dimensions of color are defined as hue, value and chroma. Color notations are frequently defined using CIELAB system developed by CIE (Commission Internationale de L’Eclairage, International Commission of Illumination), where the overall color difference attributed from all the color coordinate differences is denoted as ΔE* [26,27]. The clinical significance of color difference can be determined by perceptibility, defined as “can the color differences be seen?” and acceptability, defined as “is the difference in color acceptable?” The 50:50% perceptibility and acceptability thresholds were found to be ΔE_00_ of 1.71 and 4.00, respectively [28], which were used in this study for determining the color differences of denture PMMA. It is important to evaluate the color of PMMA with TiO_2_ film, to ensure that the esthetic outcome is clinically acceptable for patients and clinicians alike. 

Therefore, the purposes of this study were: (1) to evaluate the effect of a novel nano-ceramic coating (TiO_2_) using an ALD technique on the color surface of PMMA material, and (2) to evaluate the patients’ and clinicians’ perception and acceptance of the PMMA color with TiO_2_ coating. The first null hypothesis was that TiO_2_ coating would have no effect on the color of PMMA denture base materials. The second null hypothesis tested was that the color difference between coated TiO_2_ and noncoated PMMA would be similar to the established perceptibility threshold. 

## 2. Materials and Methods

**Figure 1:**
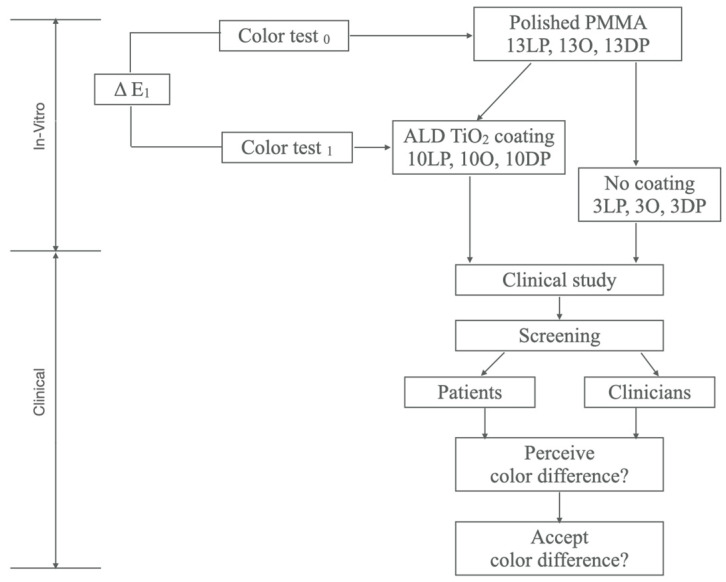
Schematic of study design used.

The experimental design and methodology were approved by the Institutional Review Board (UIC IRB Protocol #2019-0648). In this study, in-vitro and clinical approaches were performed (Figure 1). 

### 2.1. PMMA Specimen Fabrication

Thirty-nine square-shaped (10 × 10 × 2 mm) specimens of PMMA denture base acrylic resin were fabricated (Lucitone 199^®^, DENTSPLY Intl) according to the manufacturer’s protocol. Three different shades of the PMMAs, light pink (n = 13), original (n = 13), and dark pink (n = 13) were used. The polishing protocol followed that of the previous study [23]. PMMA specimens were serially polished using an ECOMET Polisher/Grinder with silicon carbide grinding paper from grit P800 to P4000. PMMA specimens were then pre-cleaned in 5% NaOH solution for 10 min, ultrasonic cleaned for 1 h, then dried by nitrogen gas. 

### 2.2. TiO_2_ Coating on PMMA Specimens

**Figure 2:**
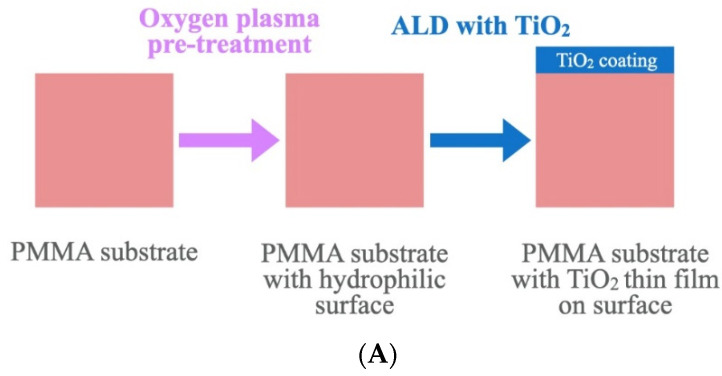

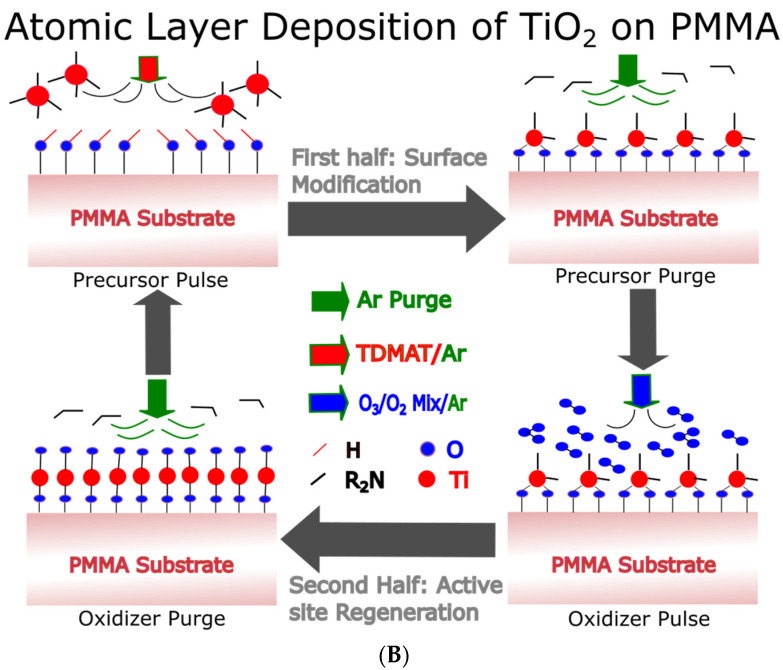
(**A**)**.** Nano-coating of PMMA specimen by Atomic Layer Deposition. (**B**). Schematic of ALD reaction on O-plasma treated PMMA using TDMAT and O_3_/O_2_ mixture.

Thirty of the PMMA specimens, 10 from each shade, were randomly selected and subjected to the TiO_2_ nano thin film coating technique. Prior to each deposition, PMMA specimens were cleaned and underwent an oxygen plasma treatment, a process summarized in Figure 2A. This was followed by ALD of TiO_2_ on PMMA. A silicon wafer was used alongside to study the growth rate. Nine of the PMMA specimens (three from each shade) did not receive any TiO_2_ coating.

#### 2.2.1. The Atomic Layer Deposition (ALD) Process

A schematic of this ALD process is described in Figure 2B. ALD of TiO_2_ was performed in a custom-made tubular, hot wall ALD reactor [29]. The reactor can be heated up to 600 °C and its base pressure is about 10 mTorr. This reactor has 4 precursor delivery lines and can deliver four different types of oxidizers: ozone/oxygen mixture, oxygen, water vapor, and small molecular weight alcohols. During the deposition, the reactor and precursor were kept at 65 °C while the delivery line in between bubbler and reactor was kept 20–30 °C higher than the bubbler temperature to prevent condensation of precursor before it reaches the reactor. The deposition chamber was kept at 500 mTorr during deposition. Tetrakis(dimethylamido)titanium (TDMAT, Sigma Aldrich, 99.999%, St. Louis, MO, USA) and ozone/oxygen mixture (1000 ppm O_3_ generated just upstream the ALD chamber) using a custom-made UV lamp system) were used as precursor and oxidizer, respectively. The precursor and the oxidizer were introduced sequentially into the reactor using computer controlled pneumatic valves. Argon (99.999%, Praxair, Danbury, CT, USA) was used as precursor carrier gas and purging gas.

#### 2.2.2. Coating Parameters and Post-Deposition Characterization

One ALD deposition cycle consisted of one 0.5 s of TDMAT pulse, 10 s of Ar purge, 1 s of ozone pulse, and 15 s of Ar purge. Silicon wafer (WaferPro, Santa Clara, CA, USA) was used to measure the post-deposition thickness using spectroscopic ellipsometry (SE) (Model M-44, J.A. Woollam Co., Lincoln, NE, USA). XPS (Kratos AXIS-165, Kratos Analytical Ltd., Manchester, UK) was performed on a single PMMA substrate after the coating process.

### 2.3. Spectrophotometric Analysis (In-Vitro Study)

The color changes were assessed using a spectroradiometer (PR 650; PhotoResearch Inc) with an optical configuration of 45-degree illumination and 0 degree observer, *before* (color test_0_) and *after* ALD coating (color test_1_). The use of the PR 650 for color research showed that ΔL*, Δa*, and Δb* did not have significant bias between the measured ceramic specimens and industrial standard (DC color checker) [30]. Spectrophotometric measurements converted the spectral data to CIELAB values with 2 degree observer and D65 lighting condition, of the color *before* and *after* the coating. Color difference (ΔE_00_) *before* and *after* coating was calculated using the CIEDE2000 formula. Factors K_L_, K_C_, and K_H_ were adjusted to 1. The mean color difference and standard deviations were calculated. Data were analyzed using Kruskal–Wallis tests to compare the differences among the three acrylic resin groups (α < 0.05). 

### 2.4. Color Perceptibility and Acceptibility (Clinical Study)

**Figure 3:**
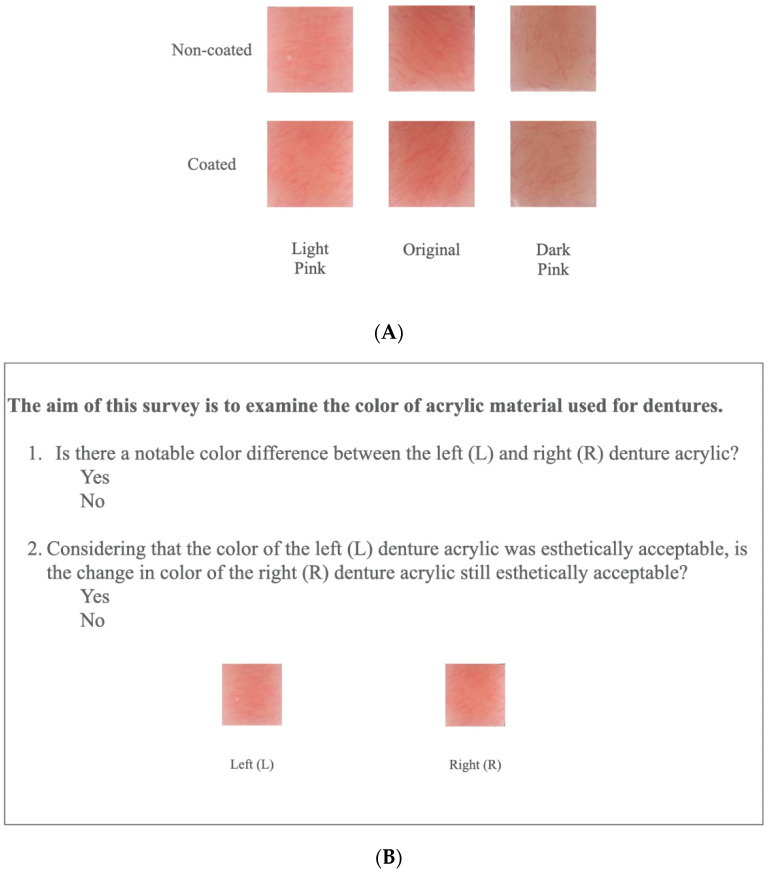
(**A**)**.** PMMA specimen fabrication. (**B**) Survey questions for patients and clinicians.

The in-vivo portion of the study evaluated the patients’ and clinicians’ perception and satisfaction of PMMA color *after* the TiO_2_ coating, and whether the color difference (ΔE_00_) of PMMA with TiO_2_ coating is different amongst different PMMA shades (light pink, original, dark pink), as shown in Figure 3A. 

#### 2.4.1. Patient Recruitment

Twenty-four participants were recruited from the University of Illinois Chicago, College of Dentistry Predoctoral and Postdoctoral Prosthodontics patient population. Patients either in active or recall status who had existing prosthesis fabricated of PMMA were recruited. Each patient was invited to complete Ishihara test voluntarily https://colormax.org/color-blind-test/, accessed on 1 October 2018. Twenty-four patients who demonstrated color proficiency (scored 100% on online test) were recruited for the study. 

The inclusion criteria were participants 18 years or older, currently using a prosthesis fabricated of PMMA, willing to participate in study, able to read and speak English, and scored 100% on the Ishihara test. 

#### 2.4.2. Clinician Recruitment

Prosthodontics faculty at the University of Illinois Chicago, College of Dentistry were invited to complete the brief online Ishihara test https://colormax.org/color-blind-test/, accessed on 1 October 2018. Ten prosthodontists certified in the American Board of Prosthodontics who demonstrated color proficiency were recruited for this study. 

#### 2.4.3. Color Survey

To permit objective analysis, the acrylic resin specimens (non-coated and coated with TiO_2_) were laid out in a frame for comparison of the perceived color differences and followed by the acceptability question (Figure 3B). An online survey was performed (Qualtrics, Provo, UT, USA). Each respondent evaluated on perception and acceptability of PMMA non-coated and coated TiO_2_ specimens. Each participant was provided with 9 different test sets comparing randomly selected non-coated and coated specimens of different shades of PMMA. Participants were instructed to stare at a gray sheet for 2–3 s between tests. The survey was conducted under standard illuminant light conditions (D55) in the clinic.

#### 2.4.4. Color Survey Analysis

Patient and clinician survey results were summed, while means and standard deviations were calculated. Comparisons by questions were made using ANOVA. Kruskal–Wallis tests and Mann–Whitney tests were performed to compare the perceptibility and acceptability of color differences (*before* and *after* coating) amongst the 3 different PMMA shades within the patients and the clinicians. All statistical analyses were performed using statistical software (IBM SPSS Statistics, v22.0; IBM Corp, Armonk, NY, USA) (α = 0.05).

## 3. Results

### 3.1. Coating Parameters and Post-Depostion Characterization

Films of TiO_2_ were successfully deposited on PMMA specimen by the ALD technique. Overall, 70 and 300 deposition cycles were performed, creating 7-nm- and 30-nm-thick TiO_2_ films, respectively. The growth per cycle for plasma treated PMMA was hence calculated to be approximately 0.1 nm/cycle.

**Figure 4:**
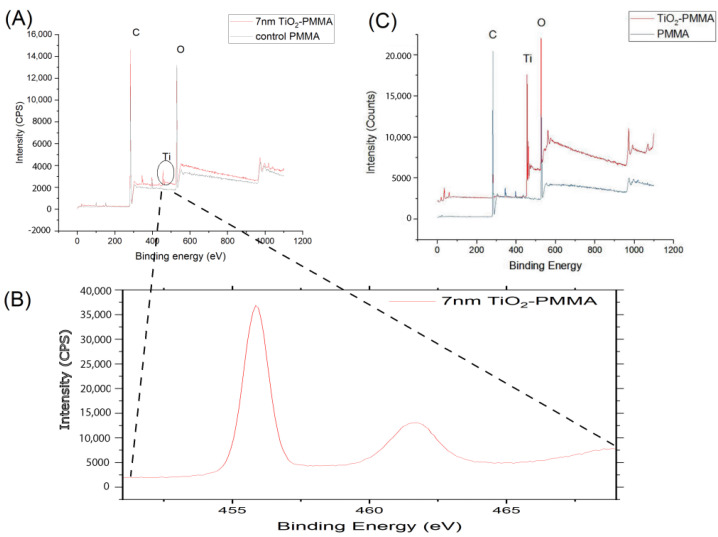
(**A**) XPS survey of PMMA before and after 7 nm ALD-TiO_2_ using the same recipe given in Section 2.2.2. (**B**). High-resolution XPS scan of 7 nm TiO_2_ coated PMMA sample between 440 to 470 eV. (**C**). XPS survey of PMMA before and after 30 nm. ALD-TiO_2_ using the same system and recipe given in Section 2.2.1 and Section 2.2.2 respectively [23].

The XPS data for a 7 nm-thick TiO_2_ film using this ALD recipe are shown in Figure 4A. High-resolution XPS for Ti peaks in the 440–470 eV range was also performed, and the corresponding spectrum is presented in Figure 4B. The data for a 30-nm-thick TiO_2_ using the same recipe and reactor were published in a prior study (Figure 4C) [23]. Therefore, after TiO_2_ ALD, Ti 2p peaks appear for both 7 nm and 30 nm coated TiO_2_. The intensity of Ti 2p also is representative of the amount of Ti on the PMMA. This peak in Figure 4A is lower than the peak observed in Figure 4C, which may be due to the lower amount of titanium in a 7-nm film as compared to the thicker 30-nm film. For color analysis (both in-vitro and clinical), PMMA coated with 30 nm film of TiO_2_ was used.

### 3.2. Spectrophotometric Analysis

Color changes were observed after the deposition of TiO_2_ coating on all three shade groups, light (ΔE_00_ = 3.2 ± 0.8), original (ΔE_00_ = 3.1 ± 0.7), and dark pink (ΔE_00_ = 3.3 ± 1.2), with no significant difference among the three shade groups (*p* = 0.845). The ΔE_00_ of light (*p* < 0.001), original (*p* < 0.001), and dark pink (*p* = 0.002) acrylic resin was significantly higher when compared with the established 50:50% perceptibility threshold (ΔE_00_ = 1.7). ΔE_00_ values of all three shades were less than the established 50:50% acceptability threshold of ΔE_00_ = 4.00 for light pink (*p* = 0.01) and original (*p* = 0.004) specimens.

### 3.3. Clinical Survey Analysis

The total number of patients and clinicians completed the clinical survey was 24 and 10, respectively.

#### 3.3.1. Perceptibility of Patients and Clinicians (Figure 5A,B)

The percentage of patients that perceived a color difference after TiO_2_ coating was 83.3%, 63.9%, and 77.8% for light, original, and dark pink, respectively, with significant difference among 3 PMMA shades (*p* = 0.022). The light shade had the highest perceived difference, whereas the original shade had the lowest perceived difference amongst the patients (*p* = 0.008). The percentage of clinicians that perceived a color difference after the TiO_2_ coating was 93.3%, 100%, and 86.7% for light, original and dark pink, respectively, with no significant difference among the three PMMA shades (*p* = 0.120). The percentages of clinicians that perceived a color difference in PMMA with TiO_2_ coating were generally higher than the patients.

**Figure 5:**
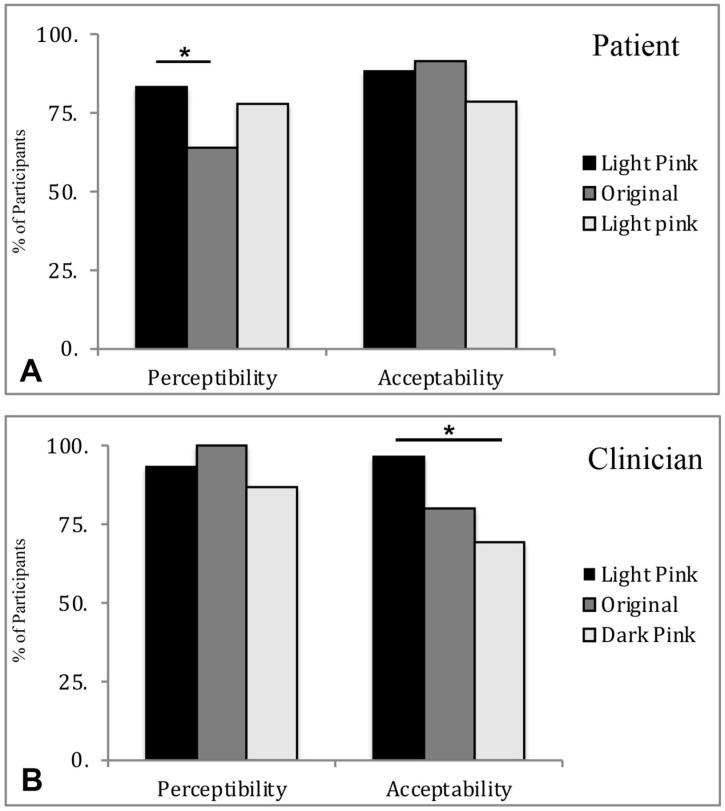
Percentage of survey participants that perceived and accepted color difference between non-coated and coated PMMA: (**A**) Patients (**B**) Clinicians. * denotes significant difference between the groups.

#### 3.3.2. Acceptability of Patients and Clinicians (Figure 5A,B)

The percentages of patients that were satisfied with the color difference after the TiO_2_ coating were 88.3%, 91.3%, and 78.6% for light, original, and dark pink, respectively; with no significant different among these three shades (*p* = 0.147). The original shade had the highest acceptance, whereas the dark shade had the lowest, amongst the patient participants. The percentages of clinicians that were satisfied with the color difference after the TiO_2_ coating were 96.4%, 80%, and 69.2%, for light, original, and dark pink, respectively; with a significant difference among the three shades (*p* = 0.032). The light pink shade had the highest acceptance, whereas the dark pink shade had the lowest acceptance, amongst the clinicians (*p* = 0.008).

## 4. Discussion

ALD is an effective technique to produce a pin-hole free, conformal films on substrate surfaces; such films can act as a diffusion barrier between the implant material and external contaminants or surface functionalization of biomaterials [23,31,32]. In this study, ALD was used to successfully deposit 30-nm-thick TiO_2_ on PMMA at low substrate temperature. 

The deposition of TiO_2_ showed color changes on acrylic denture base specimens based on spectrophotometric analysis. Therefore, the first null hypothesis was rejected. In this study, the color change between TiO_2_ coated and noncoated specimens was found to be significantly higher than the established 50:50% perceptibility threshold for acrylic denture base materials (ΔE_00_ = 1.7), which shows that there is a perceivable color difference when TiO_2_ coating of 30 nm thickness was applied. Therefore, the second null hypothesis was rejected. However, the ΔE_00_ values of all three shades were less than the established acceptability threshold (ΔE_00_ = 4.00), which demonstrate that although a color difference is perceived, it is within range of acceptance. This supports that the color change from TiO_2_ coating in this study is perceivable, but not clinically significant [12]. Other methods of applying TiO_2_ on PMMA have shown to maintain the color of the denture base materials, but increased the level of glossiness [24]. The influence of the TiO_2_ coating on the appearance of the denture base materials seems satisfactory. The findings of this study allow clinicians to make best practice decisions to use this novel application on PMMA. The TiO_2_ coating does not negatively impact the esthetics of the prostheses. TiO_2_ application on PMMA can improve hydrophilic surface properties, reduce biofilm formation, and improve the cleanability and wear resistance of PMMA [23,24,33,34]. Ultimately, the addition of TiO_2_ coating may provide positive clinical outcomes and increased patient satisfaction. 

The majority of survey participants perceived a color difference with TiO_2_ coating. The percentages of clinicians that perceived a color difference in PMMA with TiO_2_ coating were generally higher than the patients. The discrepancy in color perception between patients and clinicians has been noted in previous studies [35]. Dental professions tend to perceive more in a color discrepancy than the layperson because their professional training and experiences [35,36,37]. Similarly, professional dental experience has been reported to be directly associated with better color perception [38].

This study found that more participants perceived color differences in lighter compared with the darker acrylic resin shade. Another study also showed that human subjects were less sensitive to darker shade color differences compare with the lighter shade [39]. This may also be because some participants are more sensitive to color discrepancies in different regions of CIELAB color space [28]. Many factors affect the color perception, such as the ambient conditions including, light source, wall color, amount of light, patient’s clothing and makeup, and angulation of object [38]. In this study, the survey was conducted in the clinics under fluorescent light. The light source and environment may have a potential influence on the color perception of participants.

Overall, patients demonstrated a higher acceptance rate compared with the clinicians, except for the light pink shade, which is consistent with other studies [36,40]. Laypeople tended to have a more forgiving assessment when accepting an esthetic outcome [40]. Some suggested that laypeople had inconsistent criteria and preferences of esthetic ideals [41]. This implies that the dental professionals and the patients may have different perspectives in regard to esthetic consideration and the acceptance of dental prosthesis. In a clinical setting, a clear communication of esthetic expectation between the patients and clinicians should be established. 

Prostheses fabricated in PMMA are subjected to a multitude of intra-oral conditions, including exposure to a variety of solids and liquids, as well as multiple cleaning cycles at home or in the office. Exposure to different beverages has showed staining and color changes on the denture base material [42,43]. The color of denture base materials can also be affected by accelerated aging processes [44]. Among different manufacturers, Lucitone Hy-pro and Acron were least affected, while Compak-20 had the most appreciable color change and was the least color stable [44]. Maintaining color stability of the reconstruction prostheses should be the goal to improve patient satisfaction. 

There are some limitations in this study. This study used the sequential inquiry of perceiving differences and acceptance of the color differences [36]. This may impose a bias on the observer’s judgment of perception and acceptance of the color change of the PMMA. Further, this study only evaluated the effect of nano ceramic coating on the traditional PMMA denture base materials. With the emerging and advancement of dental materials, e.g., CAD/CAM block PMMA, 3D printed PEEK denture bases have shown improved mechanical, physical, and chemical properties and satisfactory esthetic outcomes [43,45,46]. Further examination of TiO_2_ application for the color stability of these new materials for clinical use is warranted to provide more clinical insight. 

Future studies should be directed to evaluate the color stability of TiO_2_ coated PMMA after the accelerated aging processes. Other properties that need to be further investigated include gloss, surface roughness, and translucency stability using the Kubelka–Munk reflectance theory which provides a reflectance model for translucent materials [47,48]. Further, *Candida albicans* infection of the oral cavity in post-treatment head and neck cancer patients is common [1,49]. Polymer-based obturators increased microorganisms adherence compared to titanium-based [1]. The addition of nano TiO_2_ on the intaglio surface of their obturators made by PMMA may reduce *Candida albicans* incorporation. A clinical study to investigate the effectiveness of TiO_2_ coating in reducing *Candida albicans* infection in head and neck cancer patients is warranted in the future.

## 5. Conclusions

This novel TiO_2_ coating via ALD on PMMA was successfully applied as confirmed by SE and XPS. The color changes of all three acrylic shades were above the established perceptibility threshold, but below the established acceptability threshold for denture base materials. The clinical survey demonstrated that in most cases a color difference was perceived but was clinically acceptable. In general, patients have lower perception and higher acceptance of changes in PMMA color than clinicians.
